# Gall-Stones and Their Surgical Treatment

**Published:** 1906-02

**Authors:** 


GALL-STONES AND THEIR SURGICAL TREATMENT. By B. G. A. Moynihan, M. S. (London), F. R. C. S., Senior Assistant Surgeon to Leeds General Infirmary, Leeds, England. Second edition, revised and enlarged. Octavo of 458 pages, beautifully illustrated. Philadelphia and London. W. B. Saunders & Company, 1905. Cloth $5.00 net; Half Morocco, $6.00 net.

The first edition of Mr. Moynihan's work on gall-stones was completely exhausted in eight months. Mr. Moynihan, by his masterly presentation of operative technic and clear, logical discussion of indications and contraindications has won an enviable place in contemporary abdominal surgery. In this edition, increased in size by some seventy pages, many additional case records have been incorporated and a number of new illustrations added. We note also the addition of a very valuable chapter—Congenital Abnormalities of the Gall-Bladder and Bile-Ducts. It is evident that the whole text has undergone a careful revision and all recent work along the line of gall-stone surgery included. Mr. Moynihan's book still holds first place in its field. The illustrations are very beautiful, especially the nine colored plates.

